# Study protocol: a pilot quasi-experimental trial of tele-rehabilitation and tele-drain care post-mastectomy

**DOI:** 10.1186/s40814-021-00776-5

**Published:** 2021-02-01

**Authors:** Miho Asano, Gerald Choon-Huat Koh, Preetha Madhukumar, Gladys Yu Hui Teng, Petrina Li Ling Liew, Saraswathi Nagalingam, May Leng Mabel Tan, Yee Sien Ng, Benita Kiat Tee Tan

**Affiliations:** 1 Saw Swee Hock School of Public Health, National University of Singapore, 12 Science Drive 2, #09-01, Singapore, 117549 Singapore; 2 Duke-NUS Breast Centre, 20 College Rd, Singapore, 169856 Singapore; 3 Department of Breast Surgery, Singapore General Hospital, 10 Hospital Blvd, Singapore, 168582 Singapore; 4 Department of Breast Surgery, National Cancer Centre Singapore, 11 Hospital Crescent, Singapore, 169610 Singapore; 5 Department of Occupational Therapy, Singapore General Hospital, 10 Hospital Blvd, Singapore, 168582 Singapore; 6 Nursing Division Singapore General Hospital, 10 Hospital Blvd, Singapore, 168582 Singapore; 7 Department of Nursing, National Cancer Centre Singapore, 11 Hospital Crescent, Singapore, 169610 Singapore; 8 Duke-Nus Medical School, National University of Singapore, 8 College Road, Singapore, 169867 Singapore; 9 Department of Rehabilitation Medicine Singapore General Hospital, 10 Hospital Blvd, 168582 Singapore, Singapore

**Keywords:** Tele-rehabilitation, Pilot trial, Breast cancer

## Abstract

**Background:**

Breast cancer is the leading cancer affecting women in Singapore. Its survivors commonly experience decline in physical function and quality of life post-mastectomy, due to their upper limb morbidity and wound issues. Rehabilitation can address the aforementioned issues. When rehabilitation is accessible and easy to adhere, it can optimize recovery.

Home-based tele-rehabilitation guided by healthcare professionals and self-managed by patients can potentially optimize the patients’ adherence to rehabilitation and recovery. With that in mind, a team of breast cancer specialists (oncologists, nurses, and therapists) in Singapore has developed one of the first tele-rehabilitation systems for local women undergoing a unilateral mastectomy. To our knowledge, no such systems have been evaluated or proven effective as a treatment option among local breast cancer patients with acute disabilities.

**Methods:**

This is a pilot quasi-experimental trial that aims to evaluate the feasibility of tele-rehabilitation and tele-drain care compared to usual care. Up to 40 patients (20 per group) will be recruited for this trial. They will be assigned to an intervention group that receives rehabilitation via a tele-rehabilitation system or a control group that receives rehabilitation in person at their clinic. The primary outcome of this trial is rehabilitation participation (i.e., the time spent on rehabilitation). The secondary outcomes are upper extremity functioning, perceived health, and quality of life.

**Discussion:**

As part of this pilot trial, patients who opt in for the tele-rehabilitation will be asked to share their experience with and thoughts on the tele-rehabilitation system. With the evidence obtained from the tele-rehabilitation patients of this trial, we will be able to improve the current system for our future trial. Further, our additional data on rehabilitation participation, physical function, and quality of life will help us design a sufficiently powered future main trial.

**Trial registration:**

The trial was approved by the National Healthcare Group’s Domain Specific Review Board (#2019/00283) and registered with www.ClinicalTrials.gov (#NCT04269967) in February 2020.

## Background

Breast cancer is the leading cancer affecting women in Singapore. It accounts for approximately 30% of cancer diagnosis among women in the nation [1]. The majority of breast cancer diagnoses in Singapore are at stage 1 and 2 with a high 5-year survival rate [1]. Treatment recommendations for breast cancer depend on various factors (e.g., stage of the cancer, size of the tumor, and personal preferences). In early stage of breast cancer, surgical procedure (such as lumpectomy or mastectomy) is often the first treatment option to be recommended. Further, women may elect to undergo mastectomy for different reasons (such as their hesitance toward radiation therapy and fear of recurrence) [2, 3]. Rates of mastectomy ranged from 43 to 59% locally between 2001 and 2010 [4]. Most breast cancer survivors experience decline in physical function and quality of life (QoL) due to their upper limb morbidity and wound issues after mastectomy [5].

Rehabilitation (e.g., physical and occupational therapy) is known to maintain or improve physical function and QoL [6]. It can address common issues (e.g., lymphedema, decreased endurance, and joint stiffness) that breast cancer survivors may experience after their surgery. For example, individuals with primary carcinoma who participated in post-surgery rehabilitation in a non-randomized controlled trial showed a significant improvement in various functional outcomes as compared to those who did not participate in any rehabilitation [7]. The outcomes included physical, cognitive, and social functions [7]. The current post-surgery care for breast cancer patients in Singapore includes arm exercises, arm and hand care, and self-arm massage [8].

When rehabilitation (services) is accessible and easy to adhere, it can optimize patients’ recovery post-surgery. However, a Singaporean mixed methods study of 70 patients discharged from inpatient rehabilitation found their common intention to discontinue their rehabilitation after their hospital discharge due to foreseeable barriers (e.g., accessibility, cost, and inconvenience), while the majority acknowledged the benefits of rehabilitation [9].

Telemedicine (or telehealth) refers to the use of telecommunications and technology to deliver health services (e.g., rehabilitation) outside of traditional settings (e.g., patients’ home) [10]. Tele-rehabilitation (a home-based telehealth service) guided by healthcare professionals and self-managed by patients can potentially provide a solution to the aforementioned barriers, which may lead to improved participation in rehabilitation after hospital discharge [11]. A few telemedicine studies reported that individuals who were diagnosed with breast cancer and received their rehabilitation using telecommunications and technology showed an equal or a higher amount of improvement in their health status compared to those who received their rehabilitation in a traditional way [12–14].

### Aims

The first aim of this study is to gather preliminary evidence for the feasibility of a tele-rehabilitation and tele-drain care among women who undergo a unilateral mastectomy for breast cancer. The second aim is to understand the lived experience of breast cancer diagnosis, treatment, and recovery process.

## Methods

### Study design

This is a pilot quasi-experimental trial. Patients will be placed into the intervention group (tele-rehabilitation) or control group (usual rehabilitation care) based on their preference. The total length of the study period will be 15 weeks; patients will undergo a 3-week drain care period (post-surgery) which will be followed by a 12-week rehabilitation period. A baseline assessment will be conducted prior to commencing rehabilitation (approximately 3 weeks from the surgery) and a follow-up (exit) assessment will be conducted at the end of their rehabilitation period (approximately 12 weeks from the baseline assessment). An additional qualitative study is embedded within the trial to gain a deeper understanding of the lived experience of women who are diagnosed with breast cancer to achieve the second aim.

### Patients (participants)

We plan to recruit up to 40 patients (20 per group) from the Singapore General Hospital (SGH) and National Cancer Centre Singapore (NCCS), where they will be receiving preoperative, surgical, postoperative, and rehabilitation care. Patients are recruited from multiple clinics, under a single healthcare system in Singapore, consisting of a multi-ethnic Asian population.

Patients are eligible to participate in the trial when they meet the following four criteria: (i) female 21 years of age and older, (ii) undergoing or underwent a recent unilateral breast surgery (wide excision or simple mastectomy) with lymph nodes removed, (iii) speak and write English or Mandarin, and (iv) no past or active psychiatric condition as assessed by a referring clinician. Patients are ineligible to participate if they meet one of the following four criteria: (i) having breast reconstruction, (ii) having legal blindness or severe visual impairment, (iii) having life expectancy of less than 3 months, or (iv) having previous upper limb injury or conditions that limit upper limb range of motion (shoulder flexion (< 150°) or elbow extension/flexion (< 0/145°) respectively).

### Procedure (recruitment, allocation, and data collection)

Potential participants will be approached by a breast care nurse and/or doctor (who will be providing the patient care related to breast cancer and mastectomy) during their pre-operation counseling session. These nurses and doctors will provide their patients with our study invitation letter. The patients will be asked to contact the research team directly using the contact information provided in the study invitation letter if they are interested in the study. Alternatively, if the patients agree, the nurses and doctors will obtain patients’ contact information (i.e., first or last name with an email address or a phone number only) which will be forwarded to the research team to initiate contact.

If patients agree to participate in the trial, they will complete the informed consent process with a trained member of the research team. The informed consent process can occur anytime between the time of their surgical consultation and 5 days post-surgery. After the informed consent process is completed, patients will self-assign themselves to the intervention group (tele-rehabilitation) or the control group (usual rehabilitation). The prescription and administration of drain care (education) and rehabilitation for both groups of patients are determined and managed by an assigned nurse and therapist per usual practice.

**Fig. 1:**
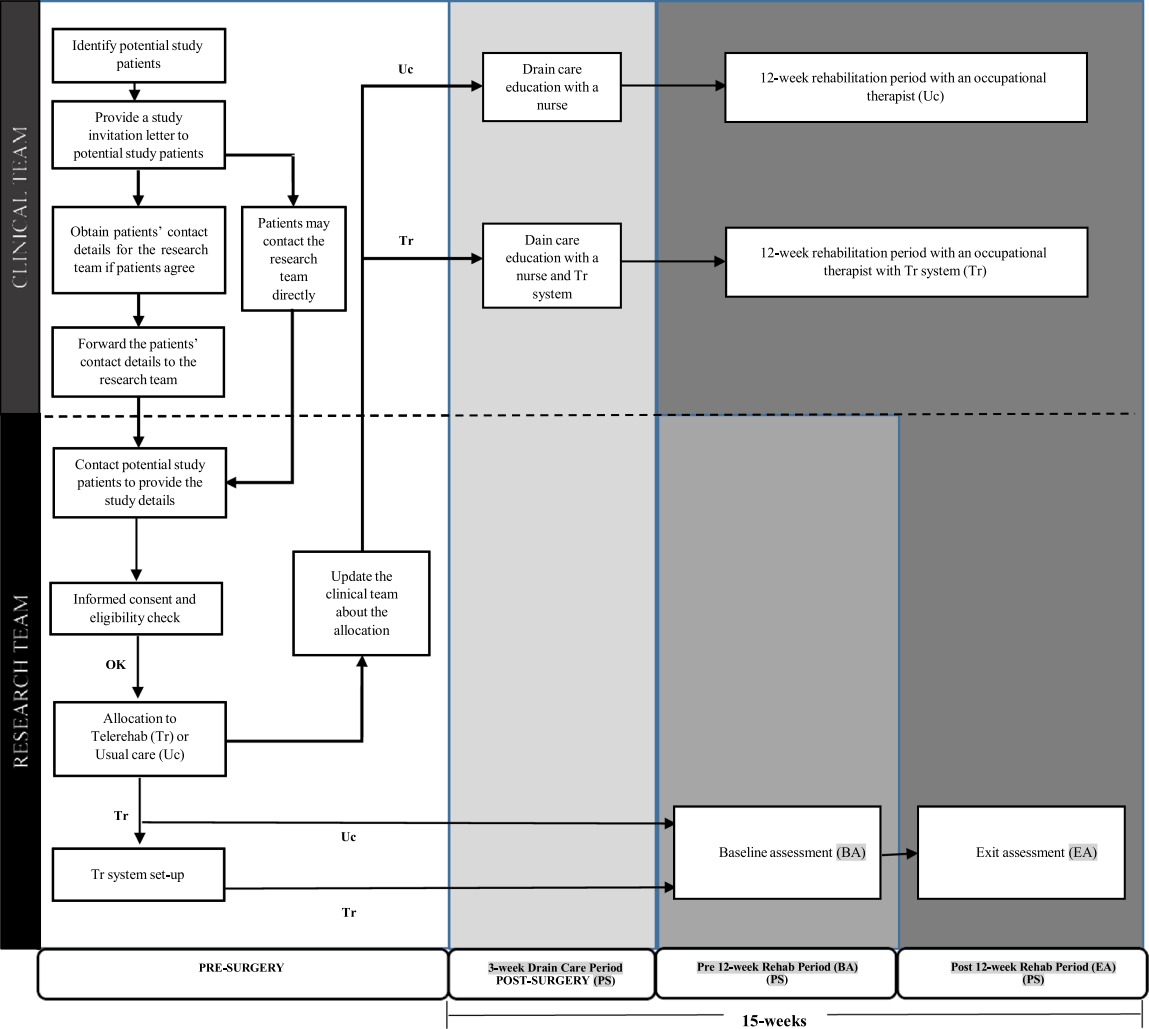
An overview of the study

All consented patients will be asked to complete two assessments with a trained member of the research team at their home (or at the university where the research is being conducted). The first (baseline) assessment will take place approximately 3-week post-surgery after completing drain-care (education) period (i.e., pre-rehabilitation assessment). The second (exit) assessment will take place after completing a 12-week physical rehabilitation period (i.e., post-rehabilitation assessment) (refer to Fig. 1 for an overview of the study outlining the timing of baseline and exit assessments). No patients, care providers, assessors, or analysts will be blinded. Any adverse events, although unanticipated, will be discussed within the team and reported to the National Healthcare Group Domain Specific Review Board within seven calendar days.

### Tele-rehabilitation (intervention) and usual care (control)

Patients in the intervention group will have the tele-rehabilitation system for up to 15 weeks. The first 3 weeks will be for drain care video education (post-mastectomy) and the following 12 weeks will be for physical tele-rehabilitation (including shoulder and arm exercise, and self-arm massage). During the physical rehabilitation period, patients (in the tele-rehabilitation group) and/or an assigned occupational therapist may arrange up to five 30-min video conferences (the number of sessions will be determined by an assigned therapist, as per request or per need of each patient).

Patients in the control group will receive the same instructions (for drain care education as well as shoulder and arm exercise and self-arm massage) by their multidisciplinary rehabilitation team but without remote support with the tele-rehabilitation and tele-drain care system. Their drain care education and physical rehabilitation sessions will instead take place in-person at their clinic with their assigned nurse and therapist (refer to Table 1 for brief descriptions of drain care education with a nurse and rehabilitation with an occupational therapist).

**Table 1:** Brief descriptions of drain care education with a nurse and rehabilitation with an occupational therapist

Drain Care Education	Arm and Shoulder Massage	Exercise
Drain care education provides information on how to manage the drainage devices and fluids collected post-mastectomy. These include proper and safe physical handling of the drainage devices, and proper sequences to empty, measure, and dispose fluids from the drainage devices.	Arm and shoulder massage are performed with the aim of managing edema (reducing arm swelling) post-mastectomy. They consist of a series of actions to facilitate pushing fluid out of the affected areas back into the bloodstream, therefore preventing fluid accumulation and limb swelling.	The exercises are performed with the aim of maintaining the active range of motion of the upper limbs, which concurrently helps to drain fluid. The exercises involve deep breathing and a series of movements including shoulder elevation, retraction, protraction and flexion, and elbow flexion and extension.
**Sample Instructions**		
Step 1. Wash your hand Step 2. Prepare your requisites Step 3. Drain fluid for measurement Step 4. Measure and record the amount of fluid drained, before disposal (twice a day)	Step 1. Take a deep breath Step 2. Place your affected arm on your shoulder with your elbow pointing to your side Step 3. Using the other hand, place it below your armpit Step 4. Gently sweep downwards past your waistline Step 5. Please ensure your palm has good contact with your body as you sweep downwards Step 6. Repeat this 20 times *Note. The arm/hand doing the massage is the contralateral side massaging the affected side.*	Step 1. Begin the exercise by clasping both hands and bringing them to the chest Step 2. Slowly bring both arms forward, making sure that your elbows are straight Step 3. Repeat this 5 times

### Measurements

A pilot study is conducted to test a set of feasibility objectives to warrant the viability of the main trial [15]. Based on an expected attrition or non-response rate, it is common to inflate the trial sample size for 10-20% [16]. The primary outcome of our pilot feasibility study is patients’ participation in the study and prescribed rehabilitation (i.e., the time spent on rehabilitation). It is a process assessment where both groups of patients will be asked to keep records of their participation in rehabilitation using a diary (logbook). The data on rehabilitation (e.g., the number of the rehabilitation sessions that they have and the duration of each session) will be collected once at the post-rehabilitation assessment (i.e., the study participation rate). Further, our tele-rehabilitation system will track the frequency and duration of the system use for the intervention group. The attrition rate of 20% or less (or the completion rate of 80% or above) is considered the criterion for the success of feasibility for our study.

In addition, all patients will be asked to complete a semi-structured qualitative interview about their experience with the recent diagnosis, rehabilitation, and recovery process at the end of the trial (after the 12-week rehabilitation period). For the additional qualitative interview, patients will be asked a series of simple open-ended questions which may include the following: (1) tell us about how you navigated your diagnosis, (2) how do you describe rehabilitation that you participated in over the past 12 weeks, and (3) how has your recovery process been—as expected or unexpected? The interview data will be digitally recorded with the patient’s permission and transcribed for qualitative analysis.

The secondary outcomes of this trial are upper extremity functioning, perceived health, and QoL. Upper extremity functioning will be measured by shoulder active range of motion (AROM) [17], arm circumference (AC) [18], the Quick Disabilities of Arm, Shoulder & Hand (QuickDASH) [19]. Perceived health will be measured by the EuroQoL-5D (EQ-5D-3L) [20] and QoL will be measured by the European Organization for Research and Treatment of Cancer Quality of Life Questionnaire Core 30 (EORTC QLQ-C30) [21]. For the secondary outcomes, all patients will be asked to complete all five measures with a trained member of the research team at both pre- and post-rehabilitation assessment. We hypothesize that both groups will show similar improvements across all secondary outcome measures.

#### Active range of motion

Patients will have their AROM (pain-free) measured for shoulder flexion, extension, and abduction on both arms using goniometry [17]. AROM for each action will be measured twice. The measurements per action per arm will be averaged for data analysis.

#### Mid upper arm circumference measurement

The MUAC will be specifically noted by measuring the arm circumference at the mid-point between the tip of the shoulder and tip of the elbow (acromion and olecranon process) [18]. Each measure will be taken twice on both arms by a trained member of the research team. The measurement per arm will be averaged for analysis.

#### Self-reported upper extremity disability

The QuickDASH is an 11-item questionnaire designed to assess self-reported disability of the arm, shoulder, and hand among individuals with upper extremity disorders [19]. Its total score may range from 0 (no disability) to 100 (most severe disability) [22]. The QuickDASH is reported to have good internal consistency and test-retest reliability [19, 23].

#### Perceived health

The EQ-5D-3L is a questionnaire designed to measure self-perceived health status of individuals [20]. It consists of five dimensions (i.e., mobility, self-care, usual activities, pain/discomfort, and anxiety/depression). Individuals are asked to select one of three responses for each domain (no problems, some problems, extreme problems/unable to perform) to describe their health status. The five responses are summed and converted to a health utility index value which ranges from −0.171 to 1. Higher values indicate better health status. The EQ-5D-3L has been shown to have good test-retest reliability [24].

#### QoL

The EORTC QLQ-C30 is a QoL measure specifically designed for cancer patients. It consists of 30 items which form functional scales, a global health status/QoL scale, and symptoms scales (including financial difficulty) [21]. Scores of all scales and single-item measures range from 0 to 100 [25]. For the functioning scales and global QoL scales, higher scores indicate better functioning; for the symptom scales, higher scores indicate higher symptom burden. The EORTC QLQ is reported to have acceptable test-retest reliability and good construct validity with the 36-Item Short Form Survey [26, 27].

### Analysis

For quantitative data, we will report means and standard deviations for parametric data, medians and inter-quartile ranges for non-parametric data, and numbers and percentages of all categorical variables for descriptive purposes. Further, inferential statistics (e.g., *T* test, chi-square test) will be performed to compare the outcome measures between the two groups. We will perform a random data (entry) check by the secondary data management personnel (who did not collect the data or complete the initial data entry). Missing data will not be replaced or imputed.

For the qualitative data, we will conduct inductive analysis that involves discovering patterns, themes, and categories related to the lived experience of breast cancer diagnosis, rehabilitation, and recovery process (using five steps of qualitative data analysis outlined by Miles and Huberman) [28].

### A power calculation

The data obtained from this pilot trial will be used to estimate a sufficient sample size for our future main trial. The existing literature suggests the minimum sample size of 10 to 15 participants per group [29] or 10% of a main trial’s sample size [30] to be sufficient for a pilot feasibility study. Considering such evidence, we plan to recruit a minimum of 10 and a maximum of 20 patients per group for this pilot trial.

## Discussion

Our pilot trial is designed to evaluate the feasibility of one of the first tele-rehabilitation systems for women undergoing a unilateral mastectomy developed in Singapore by a multidisciplinary team (e.g., surgery, oncology, rehabilitation, nursing, and engineering). To our knowledge, there is no published trial that assessed and proved the effectiveness of such systems as a treatment option among breast cancer patients with acute disabilities.

As part of this pilot trial, patients who opt in for the tele-rehabilitation will be asked to share their experience with and thoughts on the tele-rehabilitation system. With the evidence obtained from the tele-rehabilitation patients of this trial, we will be able to improve the current system for our future trial. Further, our additional data on rehabilitation participation, physical function, and quality of life will help us design a sufficiently powered future main trial. In the long term, if successful, we could have a safe and effective tele-rehabilitation system to implement as part of our regular healthcare services and optimize patients’ recovery in the comfort and privacy of their home as well as at their own convenience.

## Trial status

The trial obtained the institutional review board approval (#2019/00283) and registered with www.ClinicalTrials.gov (#NCT04269967) in February 2020. The study recruitment began in November 2020 and data collection is currently ongoing.

## Data Availability

This is a protocol of a pilot trial (version 1) which is in progress; therefore, no data are currently available.

## Abbreviations

AC: Arm circumference
AROM: Active range of motion
DASH: Disabilities of Arm, Shoulder, and Hand
EORTC QLQ-C30: European Organization for Research and Treatment of Cancer Quality of Life Questionnaire Core 30
EQ-5D-3 L: EuroQoL-5D-3L
MUAC: Mid upper arm circumference measurement
QoL: Quality of life
ROM: Range of motion
